# Biology and Management of Male‐Bodied Athletes in Elite Female Sports

**DOI:** 10.1002/dta.3876

**Published:** 2025-02-27

**Authors:** David J. Handelsman, Stéphane Bermon

**Affiliations:** ^1^ ANZAC Research Institute University of Sydney Sydney NSW Australia; ^2^ Andrology Department Concord Hospital Sydney NSW Australia; ^3^ Health and Science Department World Athletics Monaco Monaco; ^4^ LAMHESS Université Côte d'Azur Nice France

**Keywords:** puberty, sex, sports performance, testosterone

## Abstract

The physical advantages in elite power sports that allow men to surpass women are derived from the experience of male puberty. By creating testicular testosterone production 20–30‐fold over women at any age, sustained exposure over years to adult male testosterone concentrations produces larger and stronger muscles, bones, and the cardiorespiratory system with a higher blood hemoglobin explaining those advantages. While genetic advantages in exercise performance unrelated to sex are accepted in elite sports, adults who have experienced male puberty but have female gender identity, such as male‐to‐female transgender or intersex (XY Disorders of Sexual Development, DSD), create a category‐defeating conflict if they compete in female power sports. Transgender women seek feminization using estrogen treatment, which can suppress postpubertal endogenous testosterone but even sustained complete testosterone suppression leaves an unfair legacy of physical advantages. By contrast, XY DSDs do not seek hormonal feminization and recognize that testosterone suppression impedes their performance. Hence, understanding the biology of male‐bodied athletes with female gender identity is crucial to effective management, which is geared toward maintaining fairness and safety for typical women competing in elite female events. Such sex‐based restrictions are not required in recreational, junior, or nonprofessional sports or where physical advantages are not operative. After the IOC's controversial 2021 Framework document, a growing consensus among major international sports federations is establishing a working definition of male and female sports sex to facilitate fairness and safety in elite sports, which depend on power, strength, speed, or endurance.

## Graphical abstract

Graphical representation of the relationships of transgender and XY DSDF as male‐bodied athletes along the binary dimensions of biological sex.

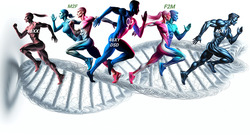

## 1 Sex, Gender and Sports Performance

Men's physical advantages in sports where power, strength, speed, and/or endurance are critical to success has traditionally led to a binary format of elite sport competition into male and female events. The performance imbalance makes a sex‐based division essential to provide a protected environment in which women have realistic chances of success in elite sport competition.

The driving mechanism for these male physical advantages is male puberty [1]. Through a 20–30‐fold increase in testosterone production over that of children and women at any age, which then persists throughput healthy adult male life, male puberty creates a cumulative effect over time of increasing muscle and bone size and strength, more powerful heart and lung function, and more hemoglobin [2] together with other less well‐characterized effects. Reconciling the binary format of sex‐based competitions with individual's sex and gender identity is straightforward for nearly all adults for whom gender identity remains consistent with their birth sex and upbringing. However, in the small minority (~0.5%) for whom adult gender identity conflicts with biological sex [3], a category‐defeating dilemma is created for participation in the binary elite sport competitions, most prominently for male‐bodied athletes with a female gender identity.

In considering this dilemma, it is necessary to make the fundamental distinction between biological sex and gender identity. Biological sex comprises multiple dimension—genetic (chromosomal), birth sex, gonadal, and hormonal sex—each representing a binary format that remains fixed throughout life. Full alignment of all these dimensions creates male and female sex in > 99.9% of individuals. Any unaligned dimension creates an intersex or disorder of sexual differentiation (DSD), the latter sometimes connoting differences of sexual development although a genetic condition making a woman unable to conceive or bear children is more than mere differences in blood group or skin, hair, or eye color.

It is sometimes considered that sex “is not quite binary”; however, this confuses inevitable, rare deviation in natural biological process with the binary process itself. For example, people are born with a heart with less than four chambers, no spleen, no gallbladder, or a single kidney—genetic disorders occurring at up to 100 times the frequency of DSDs—but these findings do not change our understanding of the characteristic features of the human body as having a four‐chambered heart, a spleen, a gallbladder, and two kidneys. Similarly, the fact that DSDs occur does not detract from the underlying biology that in humans, there are two distinct sexes.

By contrast to biological sex, gender identity is a self‐defined psychosocial construct. It does not exist at birth but develops during childhood originating from biological sex. Gender identity can be changed at will during life to differ from birth sex and childhood upbringing, just like names, language, nationality, religion, and hobbies, but unlike immutable biological features (skin, hair or eye color, blood group, genotype). Malleable variations of gender identity include opposite gender, nonbinary, gender fluid, and queer, all assuming the inherent bipolarity of gender and of sex. Crucially, an individual's gender identity can only be verified by asking that person and it is distinct from gender attraction and sexuality.

In recent times, respect for gender identity and inclusivity in all areas of life has been a salutary social change. This now allows individuals to change the recorded “sex” on public documents, such as passports and driving licenses, which may be inspected during daily life, to conform to their current adult gender identity. In that setting, however, public records of “sex” becomes an inextricable and ambiguous amalgam of sex and gender. This becomes problematic for sex on birth certificates, intended to record natal sex as a fact of birth like gestational age, date of birth, birthweight, head circumference, body length, or Apgar score—at a time when gender does not yet exist. Birth (or natal) sex is a medical diagnosis formed by visualizing the external genitalia, a highly accurate test even for untrained observers with an error rate of < 1:5000, superior to most medical tests. As a fact of birth, birth sex is not “assigned” with any discretion or arbitrariness. Hence, changing sex on birth certificates is an evasive deception attempting to rewrite birth history to better align with subsequent changes in adult gender identity. Nevertheless, however, important is inclusivity in social domains, biological sex must be ascendant over gender in at least two areas of biology—hormone‐dependent diseases (cancer, cardiovascular disease) where disease pathophysiology progresses regardless of an individual's perceptions of their gender as well as for physical performance in elite power or endurance sports.

## 2 Male Physical Advantages in Sport

The male physical advantages in sports recognize that, on average, men are taller, stronger, and faster with greater endurance than women. Although the distributional curves overlap, at extremes of elite sports, physical differences are large and significant. Male physical advantages are minimal prior to puberty (< 12 years) accruing cumulatively over time since puberty due to sustained exposure to adult male circulating testosterone concentrations [2]. That sustained androgen exposure creates larger and stronger muscles and bones, larger heart and lung capacity, and more blood hemoglobin among other less well‐characterized changes. Intuitively, these considerations long led to the traditional binary sex classification to create a protected female category. This is critically important for individual sports based on speed, strength, power, and endurance, without which women do not have a fair chance of achieving the fame and fortune from success. This has important ramifications for male‐bodied athletes with female gender identity who retain physical advantages accrued from experience of a male puberty (Table 1).

**TABLE 1:** Clinical features of biological sex.

	Male	Transgender (MTF) (untreated)	5α reductase deficiency (untreated)	Complete androgen Insensitivity	Female
Chromosomal	46 XY	46 XY	46 XY	46 XY	46 XX
Gonadal	Testes	Testes	Testes	Testes	Ovaries
Gametes	Sperm	Sperm	Sperm	Sperm	Oocytes
Hormonal (Serum T)^a^	Male range	Male range	Male range	Male range	Female range
Androgen sensitivity	Full	Full	Full	Absent	Full
Internal genitalia	Testes, epididymis, vas deferens, prostate	Testes, epididymis, vas deferens, prostate	Testes, epididymis, vas deferens, vestigial prostate	Undescended testes, no vas deferens or prostate	Ovaries, fallopian tubes, uterus, vagina
Virilization at puberty	Yes	Yes	Yes	No	No
Sex phenotype after puberty	Male	Male	Male	Female	Female
External genitalia	Penis, fused scrotum	Penis, fused scrotum	Varies^b^	Short vagina, clitoris, labia	Vagina, clitoris, labia
Legal sex	Male	Varies	Varies	Female	Female
Gender identity ^c^	Varies	Female	Varies	Varies	Varies

^a^ Circulating testosterone concentration is the marker of hormonal sex. When measured by LCMS, the 95% confidence limits are for the male range 7.7 to 29.4 nmol/L and for the female range (no PCOS) 0.06 to 1.68 nmol/L. Females with PCOS may have mildly higher than female range levels, up to 4.8 nmol/L (99.99% confidence limits), for details see Handelsman DJ et al. Endocr Rev 39:803–829, 2018.

^b^ Phenotype varies, for details see Mendonca BB et al. J Steroid Biochem Mol Biol 163:206–211, 2016.

^c^ Defined as male, female, fluid, or none.

It is well established that the male physical advantages in sport arise from the sustained exposure to adult male circulating testosterone concentrations [2]. Alternative explanations for sex differences in exercise performance such as growth hormone [4] and some unspecified Y chromosome factors [5], lack credibility [6], and have been thoroughly refuted [1, 2].

## 3 Protected Categories in Sport

While sex was the original protected category in sport based on male physical advantages, other protected categories have developed equally to provide fair competition in elite individual sports. These protected categories include age groups, weight classified sports (weightlifting, rowing, combat sports [boxing, wrestling, judo, mixed martial arts]), skill (color belts in combat sports [judo, karate, ju‐jitsu, taekwondo, aikido]), and disability (Paralympics) grading. Each requires an objective criterion to define eligibility for each stratum prior to competition. These comprise data of birth (age group), pre‐match weigh‐in (weight classification), or qualification (skill, disability). Sex‐based eligibility is also an important safety consideration in collision (football) and combat (boxing, wrestling, judo, karate, taekwondo, aikido, wushu, mix martial arts) sports. However, gender identity is not a suitable eligibility criterion as it is neither fixed nor objectively verifiable.

Sports that do not rely on male physical advantages for success seem to not need sex classification for fairness or safety, even if sex classifications exist historically. These include chess and board sports, billiards/snooker, target sports, car racing, and equestrian. Chess at elite levels has developed a modified sex‐based stratification into an Open category, a renamed but widened Male category open to anyone regardless of sex or gender, and a protected Female category. Surprisingly, men still strongly dominate elite chess and scrabble competitions [7]. Some sports have mixed features, such as golf where the long game (driving) has male power advantages of power, strength, and speed [8], whereas the short game (including putting), involving hand‐eye coordination, geospatial localization, and target accuracy, may not.

Sports with advantageous physical features but without any classification are the exception, which proves the rule on physical advantages. These sports create major competitive advantages for the favored category such as tall stature for basketball, netball and volleyball, or short stature (higher power‐to‐weight ratio) for horse jockeys, gymnastics, skating, skiing, and rock climbing. In social domains outside sport, such (dis)advantage would be considered “discrimination”; however, participation in sports is voluntary and always by agreement within its set pre‐competition rules.

Finally, sex stratification is not necessary in grassroots/community/recreational or junior (typically < 12 years old) sport, allowing for mixed sex competitions. Nevertheless, elite athletes usually begin their sports career in junior competitions.

The potential role of sex‐based eligibility for team sports is a work in progress. However, a fairness justification of sex‐based eligibility for non‐contact team sports is complex and depends on how much individual players contribute to overall team success. Some mixed gender team relays are now held in Athletics and Swimming, but they highlight the stark performance differences between men and women.

## 4 What Is an Unfair Advantage?

The term “unfair” is inherently subjective, but surprisingly reasonable, convergent estimates can be made for what is fair in elite sports.

For the “winning advantage” defined as (i) winning gold versus silver, (ii) winning a medal versus missing out, and (iii) making a final or missing out, pooled estimates over multiple Olympic Athletics and swimming events show that this winning advantage is < 1% [2]. This may be too strict a definition of fairness when applied to the male physical advantages in elite sports for considering the eligibility of male‐bodied athletes with female gender identity.

Another form of unfair advantages are the physical advantages of innovative sports equipment such as the full‐body synthetic swimsuit (LZR, 2009) and running shoes (Nike Vaporfly shoes, 2022). The swimsuit was banned by World Aquatics when it created a 3% advantage over non‐users. Similarly, after the introduction of the Nike Vaporfly shoes, World Athletics modified its rules governing competition shoes but stopped short of formally banning them despite their creation of a 4% advantage in running energy efficiency [9], exceeding the previous benefits of prior conventional shoe technology of < 2% [10]. Hence, a reasonable ceiling threshold for any systematic fair advantage is <3% [1].

## 5 Magnitude and Reversibility by Testosterone Suppression of the Male Physical Advantages

Evidence on sex differences shows conclusively that for sports where speed, strength, power, and/or endurance are critical to success, men have a strong and systematic advantages in sports performance. Based on world records in running and swimming, the male advantages are 10%–12%, for jumping 20% and for throwing events at least 20% (based on distances thrown even using heavier male implements), whereas, for certain specific events or actions, cricket bowling, baseball pitching, and golf driving, there is at least a 20%–30% advantage and that reaches over 100% for explosive punching, yanking, and pulling actions [11]. These male physical advantages become evident in early male puberty when in world age group records, 14–15‐year‐old boys (whose circulating testosterone is ~11 nmol/L vs adult males 18 nmol/L) who already surpass female world records (see Table 3 in Hilton and Lundberg [11]). After male puberty, in every year, hundreds or thousands of men surpass female athletic world records.

There is limited available evidence on how effective the complete postpubertal suppression of testosterone is to eliminate the physical advantages of male puberty. Suppression of testosterone by sustained exposure to exogenous estrogens can fully suppress endogenous testosterone secretion to the same extent as orchidectomy. A summary analysis of 13 physical measures from 10 studies measuring the degree of suppression of male physical advantages after at least 1 year of complete testosterone suppression by estrogen medication [11] showed an overall 15% decrease in the magnitude of the male physical advantages, leaving 85% of the original physical advantages. Additional studies have found analogous incomplete to minimal reversibility of male physical advantages for up to 3 years of estrogen treatment of transgender women [12]. It most likely that, even with complete suppression of testosterone for several years, such reversibility will remain far from complete, leaving legacy male physical advantages making it unfair for such individuals to compete in elite female events against typical females.

## 6 Male‐Bodied Athletes—46XY DSD

46XY DSDs are rare intersex disorders, characteristically undervirilized males due to defective gonadal development and/or androgen action, with an average community prevalence of about 1:20,000 but with geographical inhomogeneity reflecting local genetic founder effects. However, 46XY DSDs are strikingly (140–200 times) more prevalent in elite female sports than in the general community [13, 14]. This contrasts with the sport participation rates of transgender individuals reportedly low in Anglophone countries [15, 16] but not in Spain [17], but the limited studies have not reported an age‐matched non‐transgender community population.

The key 46XY DSD disorders are (a) complete or partial androgen insensitivity syndrome, (b) 5α reductase type 2 deficiency, (c) 17β hydroxysteroid dehydrogenase type 3 deficiency, and (d) various mixed gonadal dysgenesis syndromes, the latter representing genetic mutations of gonadal development yet to be fully characterized. These 46XY DSD conditions of undervirilized males have in common ambiguous or female external genitalia (depending on severity of defective virilization) at birth with undescended testes and adult male circulating testosterone concentrations from adolescence onwards. All except CAIS experience characteristic physical advantages arising from male puberty.

Although the modern standard of care for androgen sensitive XY DSD is a male sex of upbringing, in the past, especially in resource poor settings with minimal neonatal specialist care, misdiagnosis of ambiguous genitalia at birth (with or without gonadectomy) led XY DSD neonates to have a female upbringing. In such older cases, ~50% spontaneously switch to male gender identity in adolescence [18, 19], a phenomenon that now largely avoided by earlier diagnosis leading to a male upbringing [20].

It is sometimes mistakenly argued that athletes born with genetic advantages such as large hands, feet, limb, wingspan, or other genetic advantages are accepted as fair in elite sports—so why not genetic mutations causing XY DSDs? Those somatic genetic advantages occur randomly in both men and women (see for example Table 2 listing top male and female athletes with exceptional body dimensions) so they are neutral when it comes to the binary of sex‐classified events although they would not be fair if sports were classified based on limb dimensions. This differs from genetic‐based hormonal features of XY DSD, which are category‐defeating for the protected category of elite female events, as would be the case for an adult in an underage event, a middleweight in a lightweight event, or an able‐bodied athlete in a disability event.

**TABLE 2:** Typical and top‐level swimmers' body dimensions.

	Wingspan (cm)	Height (cm)	Shoe size
Typical male	179	174	10.5
Typical female	160.5	161	8.5
Missy Franklin	193	185	13
Michael Phelps	203	193	14
Ian Thorpe	190	196	17

## 7 The Evolution of Transgender and Intersex Regulations in Elite Sport

The key issue is the balance between fairness (and safety) versus inclusivity for participation in elite female sports. The IOC had an exemplary leadership role in antidoping by prohibiting androgens and other ergogenic drugs by the 1970s and developing the World Anti‐Doping Agency (WADA) in 1999; however, the IOC's record in transgender and intersex regulation has been unstable.

The IOC's first transgender regulations were the 2004 Stockholm Consensus, which enshrined the then‐contemporary Swedish approach allowing transgender woman athletes to compete in elite female events if they had surgical and legal sex change for at least 2 years. Over time, these requirements were considered unduly intrusive. In 2015, the IOC rescinded the Stockholm Consensus substituting a requirement to suppress serum testosterone to <10 nmol/L for 12 months. These arbitrary criteria lacked any consideration of accurate measurement or scientific basis for the threshold and were again sharply criticized. Ultimately, in 2021, the IOC introduced its controversial “Framework” [21], which, inter alia, insisted International Sporting Federations should set their own eligibility rules, fully favored inclusivity over fairness or safety, denied that male sex necessarily confers any sporting advantage and discouraging reliance on testosterone measurement for eligibility. That IOC Framework was strongly rejected by international sporting federations [22], sports medicine doctors [23], and top athletes (those not deterred by name‐calling abuse) as unscientific and failing to support the primacy of fairness in elite women's sport competition.

In the 2024 Olympics, unique circumstances had the IOC itself operating Olympic boxing competition bypassing its international governing federation in contrast to all other Olympic sports where the international sport governing federations conduct the Olympic sport [24]. In this unique instance, the IOC applied its unscientific “Framework” whereby the sole entry criterion for female boxing events was sex recorded on a passport, a dangerous misjudgment for a combat sport. Two 46XY DSD boxers, who had previously been excluded as ineligible by the boxing federation's medical commission, were then allowed by the IOC to participate [24]. Strikingly, androgen‐sensitive males have a nearly three‐fold stronger punch force than age‐matched females [25], illustrating the dangers as well as unfairness of male‐bodied athletes such as unmodified 46XY DSD, competing in elite female combat sports.

## 8 Criteria for Defining Sport sex

Following the IOC's abdication of leadership, major international federations have, or are, developing their own gender eligibility regulations. Beyond the unsatisfactory option of an eligibility regulation based on malleable public document “sex,” the international federations are left with four options (Table 3). The first, based on genetic sex, is based on the presence of the Y chromosome encoding the ***SRY*** gene, which induces a testis from the indifferent gonad in embryonic life thereby leading to testis development, male puberty, and adult male circulating testosterone concentrations. Its limitation is that it would fail, giving false positive for male sport sex, where an individual has undergone gonadectomy prior to puberty, has had transgender puberty blockade, or has CAIS. The second option, birth sex based on a birth certificate, would automatically exclude transgender women from female events and only rarely fails due to either misdiagnosis of neonatal ambiguous genitalia (false negative for male sex, 1:5000 births) or from adults changing the birth certificate record of sex. The third option, experience of male puberty, would be readily identified by a single serum testosterone in the male range in a healthy, resting adult (i.e., excluding doping and testosterone‐secreting tumors), which signifies that male puberty has occurred and is most consistent with the biology of sex differences in sports performance. This option would consider ineligible for elite female events anyone who has entered male puberty (defined as Tanner stage 2 or age 12 years, whichever is earlier) unless they have CAIS or, if androgen‐sensitive, they continuously maintain serum testosterone < 2.5 nmol/L. The fourth option, suppression of postpubertal endogenous testosterone in male‐bodied individuals (transgender, XY DSD), relies on a period of complete testosterone suppression (usually for 24 months) below a certain threshold consistent with the upper limit of serum testosterone concentrations of healthy women (2.5 nmol/L) to be declared eligible to compete in elite female events. The testosterone suppression criterion must be maintained continuously for as long as the individual seeks eligibility, which requires monitoring the frequency of which depends on the durability of the estrogen or GnRH analog product used to achieve testosterone suppression. A recent proposal to define Sport Sex (Table 4) has been published with its rationale [1].

**TABLE 3:** Potential criteria to define sport sex.

Option	Features
1. Genetic sex (Y chromosome, active ***SRY*** gene)	Easy to detect (blood sampling, PCR) Fails with neonatal/prepubertal gonadectomy, transgender pubertal blockade and CAIS
2. Birth sex	Fails if adult changes sex on birth certificate Permanent exclusion of transgender Fails for neonatal ambiguous genitalia (XY DSD, especially CAIS) with false negative rate 1:5000
3. Male puberty	Verify by adult male circulating testosterone Best aligned with biology of sex differences in sport No need to monitor suppression
4. Suppression of postpubertal endogenous testosterone	Requires complete suppression but incomplete efficacy and variable compliance Requires ongoing monitoring of suppression Feasible for transgender Difficult to implement for XY DSD

**TABLE 4:** Proposed definition of sport sex [1].

Sport sex is MALE in an androgen‐sensitive individual, aged 14 years or older, if they have: Serum testosterone >7.4 nmol/L^**a**^ OR Entered male puberty (Tanner stage 2) OR Has a testis or functional testicular tissue male birth sex plus SRY+ activity^**b**^	Sport sex is FEMALE for an individual aged 12 years or older, if they have: No criteria for male sex or complete androgen insensitivity OR Have had menarche OR Female birth sex plus SRY‐^**b**^ plus serum testosterone < 2.5 nmol/L^**a**^

*Note:* For further details, see Handelsman DJ. Toward a Robust Definition of Sport Sex. *Endocr Rev*. 2024;45:709–736.

^a^ Serum testosterone measured by a mass spectrometry–based method in a healthy resting adult.

^b^ Y chromosome or *SRY* translocated or absent with a SOX9 duplication.

An additional dimension is the requirement for safety, especially in collision (rugby, football) and combat sports, where the powerful physical impact of a male‐bodied athlete on another athlete is highly influenced by major sex differences, rendering such direct competition unfair, unsafe, and potentially dangerous. For example, the sex differences in power of punching is nearly three‐fold greater for men than age‐matched women [25]. This makes the inclusion of two unmodified XY DSD boxers in the 2024 Olympics a reckless and dangerous decision allowing them to compete and, unsurprisingly, win gold medals. This transpired because the IOC, implementing its notorious “Framework” [21] whereby sex recorded on a passport is the entry criterion for female events, overruled the boxing federation's medical commission, which had excluded them [24].

These criteria for fairness are not necessarily applicable to recreational/community, junior (typically < 12 years) sports at national or lower levels (if sports teams involved agree). Nevertheless, it should be recognized that elite champions usually begin in the junior leagues and have at some time to transfer to elite national and international competitions where more restrictive fairness regulations prevail.

Eligibility for elite female team sports is more complex and remains a work in progress. For safety, Rugby has implemented the same World Aquatics criteria and presumably other collision, and combat sports are likely to emulate this safety provision. However, fairness for non‐contact team sports depends on how influential an individual player is for overall team success. Possible additional criteria for non‐contact team sports include some or all of the following (a) sustained, complete androgen suppression; (b) exclude anyone who competed in male events in last 4 years; (c) approve teams rather than individuals with a limit on numbers of male‐bodied team members; and (d) modified format with an Open (mixed genders, redefined from Male) events plus protected Female category.

## 9 Identifying Male‐Bodied Athletes With Female Gender Identity From a Sports Governing Body Perspective

It is believed that transgender individuals have a low participation rate in sports [26] compared with non‐transgender peers although direct representative community‐based studies are not available. In any case, the usual attenuation into the elite class means that still few transgender individuals seek to compete in elite female sports. Individuals with male birth sex but adult female gender identity are usually, but not always, easily identified. Historically, despite rare instances of male‐bodied individuals competing in elite female events [27], it is not clear if these were transgender or XY DSD individuals rather than outright male imposters and other instances may not have been identified. Although some countries do not accept, and even outlaw, gender transition, in most countries, transgender individuals benefit from medical and psychological support especially in countries with a high level of economic development.

By contrast, 46XY DSD individuals with female gender identity competing in top‐level sport mostly come from countries with a previously low level of economic development where they were born in settings lacking sophisticated neonatal surveillance for clinical management of ambiguous genitalia. The modern standard of care for an androgen‐sensitive 46XY DSD neonate with ambiguous or female external genitalia is to usually encourage a male upbringing based on accurate neonatal investigations. Such male upbringing obviates the historical experience that most 46XY DSD individuals brought up with a female gender identity spontaneously switch to a male gender in adolescence. If they are androgen sensitive, they would be responsive to endogenous or exogenous androgen‐induced development of male external genitalia, albeit less than age‐matched peers due to the late recognition of the androgen deficiency (including during minipuberty). The present generation of female‐identifying 46XY DSD adults largely reflect a neonatal diagnosis of ambiguous or female external genitalia at birth that would not be replicated nowadays. Accordingly, that cohort of 46XY DSD individuals seeking to compete in elite female events is likely to diminish as a generation of more accurately diagnosed 46XY DSDs are born and grow up with male gender identity. For the existing cohort of 46XY DSDs, their lack of a formal diagnosis leaves the athletes concerned and uncertain of their status in elite sports while also posing a difficult challenge for sporting authorities on their eligibility for elite female events.

The benefits of an unrecognized male puberty to a XY DSD individual includes the accrual over years of post‐puberty of male physical advantages in sports. This renders those individuals highly competitive against typical XX females, an unfair advantage although in no sense deliberate cheating. These physical advantages of the unrecognized male puberty explain the 140–200‐fold increase in prevalence of XY DSD in elite female Olympic sports, compared with the general community [14, 28].

The identification of 46XY DSD athletes is always difficult. Although some may query their sports federations' medical departments (via their coach or team doctor), most cases are initially opened on the basis of results obtained during anti‐doping tests. For instance, World Athletics, in its Standard Operating Procedure internal document shared with the Athletics Integrity Unit, which operates independently, considers threshold values in both results from urine and serum antidoping tests (see Table 5). Nevertheless, such identifications are tightly constrained to protect the athlete's personal and health privacy and avoid the *prima facie* complaint of discrimination if identification was based on the athlete's phenotype and performance trends. The governing sports regulations stipulate that a minimum level of clinical and, above all, biological evidence must be provided before the health professional responsible for administering the regulations can open a case. These constraints create substantial risk of under identification of XY DSD individuals seeking to compete in female elite events.

**TABLE 5:** : Threshold values used for screening purpose for female athletes according to World Athletics eligibility regulations and reference range data [32].

Urine	Threshold (ng/mL)	Basis
Testosterone (ng/mL)	> 30*	Upper 95% CL 29.4
Epitestosterone (ng/mL)	> 30*	Upper 95% CL 28.5
Serum		
Testosterone ng/mL (nmol/L)	> 0.72 (> 2.5 nmol/L)**	
Androstenedione ng/mL (nmol/L)	> 2.29 (> 8 nmol/L)***	

*Note:* Data as ng/mL with (nmol/L). These criteria are defined for a Caucasian population [32] and need to recognize significant ethnic variations in urinary steroid excretion are known [33,34].

Abbreviation: CL, confidence limits.

* Based on the 97.5th percentile of typical female range [32].

** As per the World Athletics Regulations [38].

*** Typical female upper limit of normal [46].

## 10 The Benefits and Challenges of Using Doping Control Data in the Case Management of 46XY DSD Athletes

Although the legal and ethical aspects of using anti‐doping control data are debated [29], the 2021 World Anti‐Doping Code [30] includes a facilitatory Comment to Article 5.1 that “Where Testing is conducted for anti‐doping purposes, the analytical results and data may be used for other legitimate purposes under the Anti‐Doping Organization's rules.” Hence, a report from a doping control officer witnessing, during a urine sample collection process, clear signs of virilization in a female athlete (due to exogenous or endogenous origin) or, more often, the results of blood or urine tests carried out for anti‐doping purposes, represent valuable informative evidence to open an eligibility case on objective biological grounds.

This use is subject to procedural and regulatory constraints for using anti‐doping data when used to determine gender eligibility for competing in the female category. First, exchanges of information between the federation's medical department and the anti‐doping organization must take place within an established framework formalized by a jointly signed Standing Operating Procedure. Data storage and exchange should be stored on a secure server, shared solely on a need‐to‐know basis and be compliant with the European Union's General Data Protection Regulation (GDPR). Furthermore, the instruments and strategy used by the anti‐doping organization cannot be diverted from its primary use (anti‐doping) for the purposes of determining an athlete's eligibility. In practice, while the passive, post hoc use of such anti‐doping data is permitted, a sporting federation interested to determine gender eligibility cannot direct its anti‐doping organization to target and test an athlete nor use its whereabouts system for the sole purpose of obtaining data solely to obtain gender eligibility evidence.

Several scenarios of how relevant gender eligibility data may be derived from biological results to help decide on eligibility of a possible 46XY DSD athlete are illustrated. One relates to blood steroid profiles (Table 5). Elevated serum androstenedione (A4) concentration may be diagnostic for a rare 46XY DSD condition, 17β hydroxysteroid dehydrogenase type 3 deficiency (17βHSD3). A mutation of the 17βHSD3 gene, which is solely expressed in the testis, impairs the testicular conversion of androstenedione to testosterone. During fetal development, the resulting very low testosterone output creates insufficient dihydrotestosterone (DHT), the pure, potent natural androgen, which is the key androgen that induces formation of male external genitalia. When DHT is deficient, a 46XY infant may be born with ambiguous or female genitalia, depending on the severity of the genotype. At puberty, the marked increase in serum luteinizing hormone (LH) drives testicular secretion of testosterone and androstenedione, while other 17βHSD enzymes in non‐testicular tissues largely overcome the enzymatic block in testosterone production. This results in a highly increased diagnostic serum androstenedione to testosterone ratio [31], which can be identified during regular antidoping blood tests.

A second example is opening a case on the basis of an elevated urine concentration of testosterone and/or epitestosterone. A careful analysis of the athlete's urine steroid profile obtained as a part of the athlete biological passport (ABP) can provide a valid diagnostic clue for a 5α reductase type 2 (5ARD) deficiency mutation, among the most frequent 46XY DSD condition in elite women sports. Using reference ranges for urinary steroids derived from Caucasian athletes [32] need to also recognize ethnic variations in excretion rates [33, 34] although ratios remain no different according to ethnicity [34]. Some athletes competing in the female category return negative anti‐doping test results but may show an elevated urinary concentration of testosterone and/or epitestosterone and relevant ratios influenced by a 5α reductase deficiency. For example, the woman featured in Table 6 also consistently demonstrates reduced androsterone/etiocholanolone and 5a‐androstanediol/5b‐androstanediol ratios.

**TABLE 6:** Example of a 46XY athlete's urine steroid profile suggestive of 5α reductase type 2 deficiency.

	5α‐androstanediol, ng/mL	5β‐androstanediol, ng/mL	5α/5β ratio	Androsterone, ng/mL	Epitestosterone, ng/mL	Etiocholanolone, ng/mL	A/E	Testosterone, ng/mL
Reference range	15.2 (10, 20.6)	45.9 (26.5, 62.3)	0.30 (0.18, 0.52)	1470 (903, 2390)	11.2 (7.4, 16.5)	1480 (933, 2400)	1.02 (0.73, 1.43)	9.9 (7.2, 13.8)
Sample no.								
1	24	**310**	**0.08**	1800	76	3700	0.49	**85**
2	24	**230**	**0.1**	1700	54	3100	0.55	**66**
3	19	**220**	**0.09**	1500	45	2900	0.52	**42**
4	21	**250**	**0.08**	1500	91	3500	0.43	**65**
5	17	**220**	**0.08**	1400	25	2900	0.48	**47**
6	11	**130**	**0.08**	870	37	1300	0.67	**25**
7	26	**270**	**0.1**	1700	80	3700	0.46	**57**

*Note:* 5α/5b ratio: 5α‐androstanediol on 5β‐androstanediol ratio; A/E ratio: androsterone to etiocholanolone ratio. All steroid concentrations in nanogram per milliliter with unitless ratios and expressed as median (interquartile range). Abnormal values (outside 95% confidence limits) are highlighted in bold.

Although such a profile provides useful clues, it is not diagnostic so that confirmation of the diagnosis requires genetic testing for a 5ARD gene mutation. Nevertheless, experience has shown that these clues are reliably correlated with both genotype and phenotype for 5ARD. For example, using a cutoff of 0.67 for the A/E ratio showed a 100% sensitivity and 83% specificity for the identification of 5ARD mutation in adults [35]. Since the first extensive description of 5ARD in families from Dominican Republic, the 5α and 5β metabolites of testosterone, androstenedione, 11β ‐hydroxy‐androstenedione, cortisol, and corticosterone as well as their decreased ratios of pairs of 5α/5β steroids have provided valuable clues for the diagnosis of 5ARD [36]. Given the first quartile (25th percentile) is reported at 0.27 for 5α/5β ratio [32], our experience confirms that a cutoff value of 0.15 would detect 88% of our athletes with genetically confirmed 5ARD. That screening threshold is consistent with that of a 0.19 ratio reported for 5α/5β urinary metabolites of cortisol in young 5ARD patients [37].

## 11 Management of Testosterone Suppressive Regimens

Upon a confirmed diagnosis of 46XY DSD, the consequences depend on the international sporting federation's regulations. Some exclude 46XY DSD individuals as biologically comparable with untreated transgender women, whereas other international federations allow for eligibility subject to undertaking effective testosterone‐lowering treatment to (a) reduce her serum testosterone concentrations from within the typical male to within the typical female range and (b) maintain that suppression as long as she wishes to compete in female events. For example, World Athletics regulations require reducing and maintaining reduced her serum testosterone level to below 2.5 nmol/L for 24 months to become eligible for competition in the female category and for as long as she seeks eligibility [38].

The most acceptable, reversible options include hormonal treatments notably sex steroids or GnRH analogs. GnRH analogs include injectable depot superactive agonists in 1‐month or 3‐month depots [39] (e.g., leuprorelin, goserelin, triptorelin, histrelin), which work, despite their superactive status, paradoxically to suppress pituitary gonadotrophin and gonadal function but only after a transient period (3–4 weeks) of “flare” (gonadotrophin and gonadal stimulation) after commencing treatment [40]. An alternative option is more specific GnRH antagonists (degarelix, ganirelix, cetrorelix), which are immediately and more effective after injection and without a “flare” reaction [41, 42]. However, all GnRH analog treatments are expensive and not available in every country.

The simpler, cheaper, and highly effective option is the use of sex steroids, notably mixed estrogen/progestin containing oral contraceptives, which are available world‐wide. Typically, these contain 30 μg of ethinyl estradiol and 150 μg levonorgestrel or other progestins. If taken daily without interruption, they provide effective and sustained suppression of circulating testosterone concentration to < 2.5 nmol/L. Such testosterone suppression is as effective as orchidectomy and maintains circulating testosterone in the female reference range, but not to zero as is sometimes misunderstood. However, interruption of daily oral treatment, whether accidental or intentional, quickly releases the testosterone suppression.

Any acute hormonal suppression of endogenous testosterone in male‐bodied individuals may cause transient androgen deficiency withdrawal symptoms such as sweating, flushing, and mood disturbance. These symptoms are usually well controlled when estrogen‐based treatments (estradiol gel, tablets, or depot injections) are prescribed concomitantly.

Another option for complete suppression of endogenous testosterone is orchidectomy. Orchidectomy has the virtue of not requiring any long‐term monitoring of the effectiveness of testosterone suppression after a first confirmation at 6 weeks after surgery. This contrasts with all hormonal regimens that require ongoing hormonal monitoring as long as eligibility continues to be required. However, orchidectomy will produce side‐effects from androgen deficiency (flushing, lethargy, mood disturbance), which can be ameliorated by concomitant estradiol treatment. Although orchidectomy permanently eliminates sperm and testosterone production, both fertility preservation by sperm cryostorage and testosterone replacement are options available for individuals who remain ambivalent about the intended permanency of their gender affirming surgery. Nevertheless, the irreversibility and need for surgery make this option an increasingly unpopular last resort.

## 12 Monitoring Compliance With Eligibility Regulations for Testosterone Suppression

The hormonal monitoring of testosterone suppression is an integral part of the management, and its details depend on the nature of the hormonal suppression regimen chosen by the athlete. Depending on the option chosen to reduce her serum testosterone level, an appropriate biological monitoring scheme is required.

For hormonal treatment, after a stabilization period of 4 weeks, monitoring of testosterone concentrations commences to ensure maintenance of complete suppression (serum testosterone <2.5 nmol/L by LCMS measurement). The frequency of the serum testosterone monitoring depends on the athlete's suppressive regimen. Depot GnRH agonists when taken regularly would need to be monitored every 2 months and then, after a year, every 4 months with the testing on short notice, assuming that the injections are taken on schedule. Late or missing depot injections allow escape of testosterone suppression.

The most problematic monitoring is for the use of daily oral contraceptives or similar hormonal treatments due to their very short‐lasting suppressive effect. Missing or skipping medication for one or more days (voluntarily or involuntarily) can lead to rapid rebound in serum testosterone concentration, typically well above the required 2.5 nmol/L threshold for complete suppression. Such rebound may create a sanction for noncompliance (including restarting the period of pre‐event suppression to define eligibility and possible ineligibility) in failing to maintain complete suppression [38].

A critical feature of the eligibility monitoring is the accuracy of serum testosterone measurements. The blood testing requires an athlete should be in good general health and resting so that intercurrent illness and recent intensive exercise should be avoided. Blood sampling should in the fasting state in a morning, not immediately after an intensive training session, and measurement must be done with LCMS. Even though finding a suitable laboratory that offers LCMS may be challenging, this is critical because the widely available testosterone immunoassays operated by most chemical/clinical pathology laboratories are too inaccurate and unreliable when aiming to measure serum testosterone concentration in the low female range. Steroid immunoassay methodology has never been accepted in antidoping testing (28). Although such a methodology requirement may be troublesome for an athlete in some countries, it would always be possible to find a laboratory in another center that can provide this service.

## 13 Dilemmas of Male‐Bodied Athletes in Elite Female Events

Male‐bodied athletes with female gender identity create a category‐defeating conflict for female sports events. These conditions comprise (a) transwomen and (b) 46XY DSD (intersex), which have in common male genetic (XY), gonadal (testes), and hormonal (adult male testosterone) sex and undergo male puberty (Table 1). However, androgen sensitivity is decisive for eligibility in elite female events. Individuals with CAIS have male genetic and hormonal sex but experience no androgen‐mediated physical effects of male puberty and are therefore eligible for female event. The remainder of other androgen‐sensitive 46XY DSDs and transgender women are not eligible for female events.

Transgender and 46XY DSD present different challenges and public visibility. Male to female (M2F) transgenders are visible to the public but still very few in elite sports. For their own feminization goals, they take estrogen‐based gender affirming hormone therapy treatment that, if taken reliably, can fully suppress endogenous testosterone. By contrast, 46XY DSD with female gender identity are rarely visible to the public although their prevalence in the Olympics greatly exceeds that in the general community. They see no need for testosterone suppression and recognize that it detracts from their sports performance, tacitly confirming their unfair male physical advantages in the unmodified state. This response to using hormonal oral contraception differs from the 40% of typical female athletes using oral contraceptives, which have no detrimental effects on sports performance [43].

## 14 Challenges and Prospects for Regulatory and Biological Management of Transgender and 46XY DSD Individuals in Competitions

Although it is desirable that anyone can compete in sports recreationally or competitively if they wish, asserting this as a “right” comes at least with the responsibility that voluntary participation requires agreement to abide by the sport's existing rules and regulations. For example, doping with use of any prohibited substance, method or device is universally rejected as cheating by employing illicit, unfair advantages; however, not all unfair advantages are conscious or deliberate cheating.

In virtually all sex‐classified sports, participation in the protected category of female events is based on an individual's unverifiable assertion of their female gender identity. While male imposters aiming to compete unfairly in female events have rarely, if ever, been conclusively identified, throughout the 20th century, there were rare notorious instances of male‐bodied individuals (46XY DSDs or M2F transgender) who did compete in female events [27].

Future provision for such individuals remains a difficult dilemma. If they cannot compete fairly (or safely) in female events, then potential solutions depend on the prevalence of such individuals. If there were enough individuals, a third gender classification could be operationalized. However, in the foreseeable future, this is unlikely given the very few transgender athletes and the difficulty to systematically identify XY DSD individuals seeking to enter such a mixed gender open competition. If there are too few for a third category, then relabeling the Male into an Open (mixed gender) category, as has been done in Chess [7], may be an alternative; however, its acceptability remains to be determined.

Alternatively, identification of 46XY DSD seeking to compete in female events is challenging, Even though the prevalence of 46XY DSD is 140–200 time greater than in the general community, it remains difficult to diagnose and is likely to be still underrecognized. One solution would be for all individuals registering for an elite female competition to have, once in their lifetime, a blood test to measure serum testosterone and androstenedione and the ***sry*** gene by polymerase chain reaction. The steroid measurements would effectively screen for genetic steroidogenic disorders causing XY DSDs, namely, androgen insensitivity syndrome and 5α reductase 2 deficiency, which cause high (male) circulating testosterone concentrations as well as high circulating androstenedione concentrations caused by 17β hydroxysteroid dehydrogenase 3 deficiency. The ***sry*** gene will detect the gene usually on the Y chromosome that causes a functional testis to develop and can effectively substitute for a chromosomal analysis (karyotype) for these sporting purposes.

There are no known false positive for the ***sry*** pcr test other than substitution of another person's DNA. The ***sry*** gene is absent in about 1:80,000 births causing an ***sry*** negative XY DSD condition (Swyer's syndrome [44]) in which no testis develops thereby eliminating male puberty and its hormonal consequences, making them eligible for elite female competition. Exceptionally rare instances occur of ***sry*** negative XY DSDs where some testis development can occur if the function of the ***sry*** gene's protein product is replaced by a genetic duplication of the ***sry*** target receptor, SOX9 or SOX3 [45].

The blood steroid concentrations of an XY DSD is expected to be elevated into the male rather than the female range. However, unexpectedly low concentrations (in the female range) could be due to a person either gonadectomized in infancy during clinical management for ambiguous genitalia or who has been puberty‐blocked from prior to onset of male puberty. Alternatively, a doping administration of an androgen other than testosterone could be detected by a concurrent urine anti‐doping test.

This combination of hormonal steroid and genetic tests could be determined by a single blood sample, which could be as simple as a dried blood spot, which can be stored indefinitely at room temperature thereby avoiding venepuncture and need for blood sample storage. Such a once‐in‐lifetime test could reliably determine an individual's eligibility for the protected category of elite female events.

## Conflicts of Interest

SB is Medical Director of World Athletics but neither author has any conflicts of interest.

## Data Availability

Data sharing is not applicable to this article as no new data were created or analyzed in this study.
